# TIA OR ANOTHER CHAMELEON?

**DOI:** 10.51894/001c.122830

**Published:** 2024-08-30

**Authors:** Beom Ho (Daniel) Lee, Malitha Hettiarachchi

**Affiliations:** 1 College of Osteopathic Medicine Michigan State University; 2 Detroit Medical Center, Sinai-Grace Hospital

## 16

### INTRODUCTION

Transient Ischemic Attacks (TIA) are sudden focal neurological deficits of vascular origin, typically lasting less than an hour, with diverse cortical manifestations. However, diagnosing and managing the underlying condition can be challenging due to various conditions that mimic TIA symptoms.

### CASE DESCRIPTION

A 56-year-old female patient arrived at the emergency department with TIA symptoms – unilateral left-sided paresthesia and weakness in the face, upper extremity, and lower extremity. She also experienced mild retrosternal chest pain and blurry vision. Clinical examination revealed left-sided facial drooping and mydriasis of the left eye, unresponsive to light. The upper extremity had a power rating of 4/5 with intact reflexes, while the lower extremity exhibited a power rating of 3/5 with intact reflexes and a negative Babinski sign. The patient’s past medical history was significant for hypertension and multiple cerebrovascular events, beginning in 2015 and the most recent occurrence in October 2022, without any residual weakness. Despite multiple admissions for the same symptoms, the MRI, CT, and CTA consistently showed no evidence of ischemic or hemorrhagic changes after the initial cerebrovascular event in 2015. ECG and echocardiography indicated a regular sinus rhythm without atrial fibrillation, and normal ejection fraction with no evidence of ASD or PFO, respectively. With a NIH stroke scale score of 4, the patient did not receive TPA but was started on dual antiplatelet and statin therapy. The patient described fluctuations in her condition, with some neurological examinations showing a 5/5 strength in all extremities and others indicating a 4/5 weakness in the left upper and lower extremity. Subsequently, a urine drug screen returned positive for amphetamines, despite the patient’s adamant denial of cocaine or amphetamine use. Ultimately, the patient was diagnosed with a cerebrovascular event likely triggered by cocaine use.

### CONCLUSION

This case emphasizes that TIA-like symptoms may stem from other conditions, such as cocaine-induced vasospasm, underscoring the importance of reviewing medical records. When faced with abnormal presentations and/or prolonged duration of TIA symptoms, healthcare providers should consider investigating secondary causes of these symptoms.

